# 25-Hydroxyvitamin D Status and Its Association with Sleep Duration in Chinese Schoolchildren

**DOI:** 10.3390/nu10081013

**Published:** 2018-08-03

**Authors:** Qing-Hai Gong, Si-Xuan Li, Hui Li, Qi Chen, Xiao-Yong Li, Guo-Zhang Xu

**Affiliations:** 1 Ningbo Center for Disease Control and Prevention, Ningbo 315010, Zhejiang, China; gongqinghai@163.com (Q.-H.G.); lisx@nbcdc.org.cn (S.-X.L.); lih@nbcdc.org.cn (H.L.); 2 Yinzhou Center for Disease Control and Prevention, Ningbo 315100, Zhejiang, China; chenqielton@163.com (Q.C.); li_xy1115@163.com (X.-Y.L.)

**Keywords:** sleep duration, vitamin D, adolescents

## Abstract

Objective: To examine the association between 25-Hydroxyvitamin D (25(OH)D) levels and sleep duration among Chinese adolescents. Subjects and methods: A school-based cross-sectional study was conducted among Chinese adolescents in 2017. Data on a total of 800 adolescents aged 8–14 years was used for this study. Anthropometric measurements such as height and weight were measured by trained research staff. Serum 25(OH)D and lipids were measured in the laboratory. Sleep habits and other health-related behaviors were tested by questionnaire. Results: 25(OH)D levels were significantly positively correlated with sleep duration (*r* = 0.11, *p* < 0.05). In multivariate logistic regression analyses, insufficiency/deficiency of vitamin D (25(OH)D ≤ 20 ng/mL) was significantly associated with increased probability of short sleep (AOR = 1.67, 95% CI = 1.14–2.43). Conclusions: Low 25(OH)D levels were independently associated with the risk of insufficient sleep in Chinese adolescents.

## 1. Introduction

Sleep, much like diet and physical activity, is a critical indicator of health and well-being in children and adolescents [1,2]. Lack of sleep or short sleep duration may increase the risk of obesity, cardiovascular disease, diabetes mellitus, total mortality and other unhealthy conditions [3,4,5,6].Nowadays, insufficient sleep is a public health problem in children and adolescents worldwide [7,8,9]. A recent epidemiological study in Sweden found that roughly 40% of children (aged 10 years) had sleep duration of less than 9 h per day [10]. Our previous cross-sectional study also showed a similar result [7]. Vitamin D is an essential micronutrient during the growth and development of the human body, and 25-hydroxyvitamin D (25(OH)D) is the most sensitive clinical marker for vitamin D status [11].Vitamin D deficiency has been receiving increasing attention as a potential publich ealth concern around the world [12,13]. A study in UK found that 70% of adolescents aged 14.7 to 16.6 years were vitamin D deficient [14]. In China, across-sectional study in Wenzhou (28° N latitude) reported that nearly 40% school children had a 25(OH)D level <20 ng/mL [15]. Recent evidence suggested that Vitamin D deficiency was associated with an increased risk of metabolic syndrome (MetSyn) [16], obesity [17], diabetes [18], depression [19] and other health problems [20]. A few studies have reported the relationship between low vitamin D levels and short sleep duration among adults and adolescents [21,22]. However, other studies found that vitamin D level was not associated with sleep [23,24]. As far as we know, no epidemiological study regarding the association between sleep duration and 25(OH)D has been carried out in Chinese children. Thus, the purpose of the present study was to examine the relationship between 25(OH)D levels and sleep duration among schoolchildren in Ningbo, China.

## 2. Material and Methods

### 2.1. Subjects and Experimental Design

This study used data from the 2016–2018 Ningbo Youth Risk Behavior Survey (NYRBS), a school-based prospective study in Ningbo, Zhejiang province, China. The present study included a questionnaire survey and a physical examination. The study used a convenience sample of adolescents aged 8–14 years selected from three primary schools in October 2017 in the main urban region of Yinzhou District, Ningbo, Zhejiang Province, China. All participants were in apparently good health. Exclusion criteria included: hepatic or renal diseases; metabolic rickets; ongoing use of antilipemic medications or growth hormone; and any other medication that could influence vitamin D levels. Methods used for exclusion included medical history, physical examinations and laboratory tests. Overall, 818 students from 17 classes participated in the present study. However, 18 students were excluded because of illness or menstruation. Finally, the present analyses were based on 800 health students who participated in this study. The nature and scope of the population study were explained to the children and their parents (or legal guardians) who were given informed consent. The study was approved by the Ethics Committee of the Ningbo Municipal Center for Disease Control and Prevention (NO. 201606). All participants completed a questionnaire which contained demographic information, general health status, health-related behaviors and past medical history.

### 2.2. Blood Collection and Anthropometrics Measurement

Serum sample collection (5 mL) was performed between 7:30 a.m. and 8:50 a.m. by a trained technician (nurse) after 12 h of fasting. Samples were allowed to clot and then were centrifuged, separated and stored at −70 °C until they were tested. Anthropometric measurements such as height, weight, waist and hip circumferences were conducted in this study. Participants fasted the night before and anthropometric measurements were measured the next morning by trained research staff. All staff used the same devices, which were calibrated at the time of measurement. The height and weight of adolescents were measured with thin clothes (shorts and T-shirts) but with shoes removed. Height was measured to the nearest 0.1 cm with a free-standing stadiometer mounted on a rigid tripod (GMCS-I, Xindong Huateng Sports Equipment Co., Ltd., Beijing, China). Fasting body weight was measured to the nearest 0.1 kg on a digital scale (RGT-140, Weighing Apparatus Co., Ltd., Changzhou, China). BMI was calculated as weight in kilograms divided by height in meters squared (kg/m^2^). The waist circumferences (WC) and hip circumferences were measured with a circumference measurement tape (82203B, Hoechst mass, Frankfurt, Germany). Waist height ratio (WHtR) was calculated as the ratio between WC in centimeters (cm) and height in cm. Waist Hip Ratio (WHR) was calculated as the ratio between WC in cm and hip circumferences in cm. Weight, height, WC and hip circumferences were measured three times and the means were used for analyses.

### 2.3. Biochemistry Analysis

Serum 25(OH)D was measured by electrochemiluminescence immunoassay (ECLIA) method (MS-880B, Medicom, Ningbo, China). Cut-off values for serum 25(OH)D levels include: >20 ng/mL for vitamin D sufficiency, and ≤20 ng/mL for insufficiency and deficiency, based on Institute of Medicine (IOM) [25]. All of the intra- and inter-assay test coefficients of variation were <5%.

### 2.4. Sleep Duration and Healt and h-Related Behavioral Factors

In our study, all participants were asked to complete self-administered surveys during a 45-min class period in their classroom. Sleep duration was assessed by the following questions, “On an average morning, what time do you get up?”, “On an average evening, what time do you go to bed?”. All participants reported their time of going to bed and the time of arising. Sleep duration was calculated according to the following equation: sleep duration = (get up time + 24) − (go to bed time) [7,26]. According to previous epidemiology studies, sleep duration was dichotomized as insufficient sleep (<9 h/day) and sufficient sleep (≥9 h/day) for children aged 8–14 years [26,27,28,29]. Details of the questionnaire relating to health-related behaviors, including dietary, physical activity, breakfast skipping, cigarettes and alcohol use were reported in our previous study [7].

### 2.5. Statistical Analysis

Continuous variables were summarized as means (standard deviations), and categorical variables were summarized as percentages. One way analysis of variance was used for continuous variable, whereas categorical variables were analyzed using a chi-square test. The relationship between 25(OH)D levels and insufficient sleep (dependent variable) was evaluated with multivariate logistic regression model. Statistical significance was set at *α* = 0.05 and statistical analyses were performed using the Statistical Package for Social Sciences (SPSS) for Windows, version 17.0 (SPSS Inc., Chicago, IL, USA).

## 3. Results

The general characteristics of the study participants are shown in Table 1. Of the 800 children included in our study, the mean age of the participants was 11.15 ± 1.91 years, and 54.50% of participants were boys. The mean sleep duration of the children was 9.17 ± 0.97 h, the mean serum 25(OH)D concentration was 22.38 ± 6.03 ng/mL.

**Figure 1:**
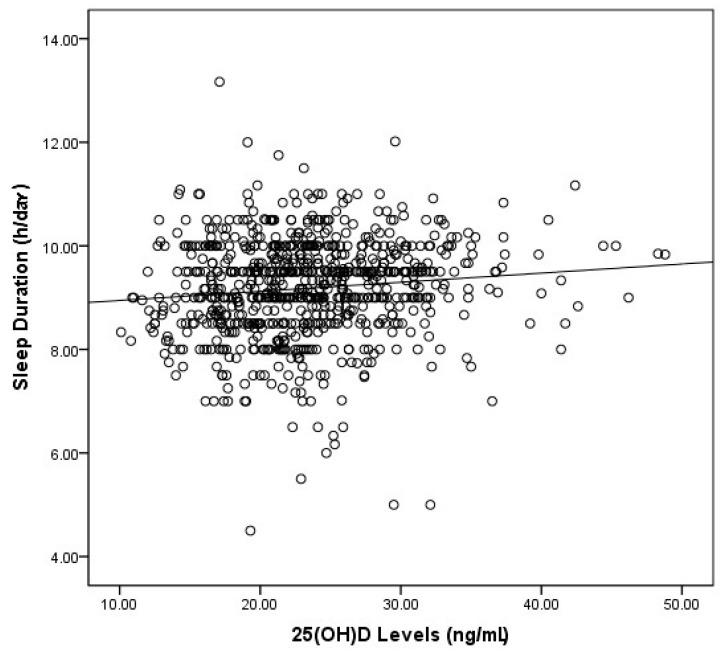
Correlation between sleep duration and 25(OH)D level in 800 subjects (*r* = 0.11; *p* < 0.05).

Table 2 shows the characteristics of the study participants according to sleep duration category. Nearly 1/3 (32.8%) of the study subjects were sleep insufficient (sleep duration <9 h per day), 30.3% of the study subjects were vitamin D insufficient and deficienct (serum 25(OH)D level ≤20 ng/mL). There were significant differences among three groups in age, parents’ highest education at college level, BMI, current drinking, breakfast skipping and 25(OH)D levels (*p* < 0.001). No difference in gender, parent’s marriage status, current smoking or physical activities were found. Figure 1 shows that there was a definite positive correlation between sleep duration and the concentration of 25(OH)D in this study (*r* = 0.11, *p* < 0.05).

The relationship between 25(OH)D levels and sleep duration was examined by multivariate logistic regression analyses (Table 3). Insufficiency and deficiency of vitamin D (25(OH)D ≤ 20 ng/mL) was significantly associated with the increased probability of insufficient sleep adjusted for age groups, gender, parents highest education at college level, breakfast skipping, current drinking, and BMI (AOR = 1.67, 95% CI = 1.14–2.43).

## 4. Discussion

Over the past century, sleep duration has decreased by 0.75 min nightly per year both in children and adolescents [30]. In our present study, we found that 32.8% of children had insufficient sleep (sleep duration <9 h/day), which was consistent with the findings of recent studies [10,19]. The prevalence of vitamin D (25(OH)D) insufficiency and deficiency in our study was a little lower than prevalence described in previous studies [31,32]. One study conducted in western China with a smaller sample (*n* = 443) found that 49% children and adolescents were vitamin D deficient (25(OH)D level ≤20 ng/mL) due to avoidance of sunshine or inadequate dietary intake of vitamin D [31], while another study conducted in Ethiopia (*n* = 174) found that 42% of the study subjects were vitamin D deficient (25(OH)D level ≤20 ng/mL) [32].However, all of these results were based on non-randomization studies with small sample sizes, and these studies lacked adequate representation of the population. Therefore, more studies with large representative samples are still needed.

Our analyses revealed that 25(OH)D insufficiency and deficiency had a significantly higher odds ratio for insufficient sleep compared with adolescents with 25(OH)D sufficiency after adjusting for confounders, which was similar to the findings of recent cross-sectional studies on adults [21,23,33]. However, there have been few researches examining the relationship between vitamin D levels and sleep duration among children and adolescents. To the best of our knowledge, this is the first study to analyze the association between serum 25(OH)D concentrations and sleep duration in young Chinese students.

To date, the mechanisms for a link between sleep duration and vitamin D are not well understood. There might be some possible reasons for explaining the relationship between vitamin D status and sleep duration. For instance, lower 25(OH)D levels might lead to sleep disorders through affecting sleep regulating substance, such as melatonin, tumor necrosis factor alpha (TNF-*α*), interleukin-1 (IL-1) and prostaglandin D2 (PD2) [16,17,18]. Another possible explanation is that vitamin D receptors have been found in specific regions in the central nervous system involved in regulating sleep, which include the anterior and posterior hypothalamus, the raphe nuclei, the midbrain central gray, and the nucleus reticularis pontis caudalis and oralis [34,35,36,37,38,39]. This explanation was supported by some intervention studies in humans, which found improved sleep duration and sleep latency with higher levels of vitamin D supplementation [40,41]. However, sleep disorders might be link to changes in dietary habits and outdoor activity’s patterns, which could affect 25(OH)D levels [42]. Thus, these relationships between sleep duration and 25(OH)D levels may be bidirectional.

This study provided new evidence that plasma 25(OH)D status was associated with sleep duration among children. The findings of this study might have important implications that 25(OH)D status is a potential biomarker of insomnia or lack of sleep in children. These findings have implications for designing prevention strategies for child sleep problems. Some limitations of the present study need to be mentioned. First, causality could not be inferred in our data due to the cross-sectional nature of study design. Second, the population of our study was not a large representative sample. Thirdly, this study may have some bias in measured behavioral factors because it is self-reported. For instance, sleep duration was calculated from reported get up time and go to bed time, which may not be accurate. Finally, we were not clear about some relevant variables such as the information on participant’s dietary habits, psychological status and sleep quality, which may also be shown to be related to sleep duration.

## 5. Conclusions

In summary, the present study revealed that vitamin D (25(OH)D) deficiency was an independent predictor of insufficient sleep among Chinese schoolchildren. The results implied that adequate vitamin D in Ningbo schoolchildren might curb sleep insufficiency problems. As the present study only highlighted the association between vitamin D deficiency and insufficient sleep duration in our subjects, prospective studies and randomized controlled trials are required to establish the causality between serum vitamin D and sleep outcomes.

## Figures and Tables

**Table 1:** Characteristics of the study subjects (*n* = 800).

Variables	
Age, years, mean (SD ^a^)	11.15 (1.91)
Gender, *n* (%)	
Male	436 (54.50)
Female	364 (45.50)
Parents’ highest education at college level, *n* (%)	
Both had college degree	283 (35.38)
Only one of them had college degree	111 (13.88)
None of them had college	406 (50.24)
Parents’ marriage status, *n* (%)	
Married	754 (94.25)
Divorced/widowed/separated	46 (5.75)
Bedtimes, mean (SD)	21:12 (0:41)
Wake times, mean (SD)	6:23 (0:36)
sleep duration, mean (SD) (h)	9.17 (0.97)
BMI ^b^, mean (SD) (kg/m^2^)	17.61 (3.19)
Hipline, mean (SD) (cm)	74.19 (14.28)
Waistline, mean (SD) (cm)	65.67 (33.03)
Waist-to-Hip Ratio (WHR), mean (SD)	0.90 (0.30)
Waist-to-Heigth Ratio (WHtR), mean (SD)	0.46 (0.26)
25(OH)D, mean (SD) (ng/mL)	22.4 (6.0)

^a^ SD, standard deviation; ^b^ BMI, body mass index; Data are presented as mean (standard deviation), or numbers (percentage).

**Table 2:** Characteristics of the study subjects by sleep duration (*n* = 800).

Variables	Sleep Duration			
	<9.0 h/day (*n* = 262)	9.0–9.9 h/day (*n* = 353)	≥10.0 h/day (*n* = 185)	*p*
Age, years, mean (SD ^a^)	12.22 (1.75)	10.24 (1.73)	9.41 (0.90)	<0.001
Age group (years), *n* (%)				<0.001
≤10	52 (19.85)	224 (63.46)	148 (80.00)	
>10	210 (80.15)	129 (36.54)	37 (20.00)	
Gender				0.071
Male, *n* (%)	128 (48.85)	205 (58.07)	103 (55.68)	
Parents’ highest education at college level, *n* (%)				<0.001
Both had college degree	61 (23.28)	136 (38.53)	86 (46.49)	
Only one of them had college degree	33 (12.60)	49 (13.88)	29 (15.68)	
None of them had college	168 (64.12)	168 (47.59)	70 (37.84)	
Parents’ marriage status, *n* (%)				0.170
Married	252 (96.18)	327 (92.63)	175 (94.59)	
Divorced/widowed/separated	10 (3.82)	26 (7.37)	10 (5.41)	
Bedtimes, mean (SD)	21:44 (0:39)	21:08 (0:23)	20:35 (0:34)	<0.001
Wake times, mean (SD)	5:53 (0:24)	6:29 (0:24)	6:54 (0:35)	<0.001
BMI ^b^, mean (SD) (kg/m^2^)	18.93 (3.58)	17.18 (2.58)	16.58 (2.57)	<0.001
25(OH)D, mean (SD) (ng/mL)	21.9 (5.7)	24.3 (5.8)	23.6 (6.2)	<0.001
25(OH)D Status, *n* (%)				<0.001
Deficiency/Insufficiency (≤20 ng/mL)	106 (40.46)	80 (22.66)	56 (30.27)	
Sufficiency (>20 ng/mL)	156 (59.54)	273 (77.34)	129 (69.73)	
Felt sad or hopeless (yes) ^c^,*n* (%)	34 (12.98)	40 (11.33)	18 (9.73)	0.564
Current smoking (yes) ^d^, *n* (%)	9 (3.44)	10 (2.83)	4 (2.16)	0.729
Current drinking (yes) ^d^,*n* (%)	34 (12.98)	24 (6.80)	5 (2.70)	<0.001
Breakfastskipping (breakfast consumption frequency ≤6 days/week), *n* (%)	48 (18.32)	27 (7.65)	6 (3.24)	<0.001
Physical activity ^e^				
Moderate physical activity (≥1 day/week), *n* (%)	254 (96.95)	338 (95.75)	178 (96.22)	0.742
Outdoor activity (≥2 days/week), *n* (%)	198 (77.95)	252 (75.68)	135 (75.00)	0.732
Watching TV (≥2 h/day), *n* (%)	44 (17.32)	51 (15.18)	29 (16.11)	0.782

^a^ SD, standard deviation; ^b^ BMI, body mass index; ^c^ During the past 12 months; ^d^ During the past 30 days; ^e^ During the past 7 days.

**Table 3:** Odds ratio (OR) and 95% confidence interval (CI) for insufficient sleep (<9 h/day) by categories of 25(OH)D levels (*n* = 800).

	25(OH)D Levels (mg/mL)		*p*-Value
Sleep Duration Insufficiency (Less Than 9 h)	≤20	>20	
Model 1	1.71 (1.17–2.49)	1	0.005
Model 2	1.07 (1.01–1.13)	1	0.007
Model 3	1.67 (1.14–2.43)	1	0.009

Model 1: Adjuated for age and gender; Model 2: Adjuated for age, gender, and BMI; Model 3: Adjuated for age, gender, parents highest education at college level, breakfast skipping, current drinking, and BMI.
